# Gastrointestinal CMV Disease and Tuberculosis in an AIDS Patient: Synergistic Interaction between Opportunistic Coinfections

**DOI:** 10.1155/2018/8047892

**Published:** 2018-06-11

**Authors:** Miriã Boaretto Teixeira Fernandes, Pedro Afonso Nogueira Moisés Cardoso, Luiza Bassani Altoé, Izana Junqueira de Castro, Guilherme Almeida Rosa da Silva, Walter de Araújo Eyer-Silva, Marcia Lyrio Sindorf, Rodrigo Panno Basílio de Oliveira, Marcelo Costa Velho Mendes de Azevedo, Jorge Francisco da Cunha Pinto

**Affiliations:** Universidade Federal do Estado do Rio de Janeiro (UNIRIO), Rio de Janeiro, RJ, Brazil

## Abstract

The AIDS pandemic has made diseases such as tuberculosis, CMV disease, and other opportunistic infections more prevalent; these diseases may even be found to be associated among themselves, and the natural history of each disease may present in an unusual manner. We report the case of a 41-year-old man with HIV (CD4 of 144 cells/dL) and HCV with hematochezia due to tuberculosis in the ileocecal valve and descending colon and CMV tissue invasive disease in the esophagus and descending colon. Coinfection among tuberculosis and cytomegalovirus in the gastrointestinal tract was described only once in a patient with a recent diagnosis of HIV that affected the distal ileum and ascending colon. We will discuss the peculiarities of the case and the behavior of the immune system in the face of simultaneous opportunistic infections. This is a challenging scenario that has scarce publications and is of great clinical importance.

## 1. Introduction

The pandemic human immunodeficiency virus (HIV) has dramatically changed the opportunistic infections epidemiology, such as for tuberculosis and cytomegalovirus (CMV) disease. Both have become more prevalent and could be found in association in the same patient, possibly with an unusual natural history [1–4]. The neglect and poor adhesion to antiretroviral therapy (ART), the therapeutic failure, and the lack of access to this therapy made possible that this type of manifestation continued to occur in the same manner it used to occur in the pre-ART era [1–4].

The highest prevalence of tuberculosis cases is concentrated in the south of Asia and Africa [5]. However, in Brazil, mostly in Rio de Janeiro, the Brazilian province with highest prevalence, the disease's incidence is approximately 70.57 in 100,000 inhabitants in 2012 [6]. The low socioeconomic condition, undiagnosed sick people in circulation, and the poor adherence to treatment result in an increase of new cases, which are often associated with HIV immunodepression [7]. It is estimated that 95% of the cases in the world occur in underdeveloped or emerging countries with lower life quality, which shows the relationship between this disease and socioeconomic issues [8]. By 2015, it was estimated that at least 11 million people were coinfected with HIV and *Mycobacterium tuberculosis* in the world, and from individuals diagnosed with tuberculosis in 2007, approximately 11% were HIV positive. Tuberculosis in the gastrointestinal tract, of interest to our case, is a less common presentation compared to the classical pulmonary form, which occurs mainly in immunosuppressed individuals [9].

Another infectious agent of interest to our study, which became more prevalent with the spread of AIDS, is cytomegalovirus (CMV), a virus from the herpesvirus family. Beyond the congenital, the perinatal, and the mononucleosis-like manifestations, cytomegalovirus is also a typically opportunistic infection. In South America, Africa, and Asia, where the prevalence of CMV infection is higher, primary infection usually occurs during childhood, following a period of clinical latency [10]. Reactivation occurs in an immunosuppressive situation. CMV promotes vasculitis and inflammatory lesions in several tissues, such as the retina, lung, and gastrointestinal tract, especially in HIV patients with CD4 T cells <50 cells/dL [11, 12].

A relevant topic is that the simultaneous manifestations of opportunistic diseases in the context of AIDS are recurrent, reaching up to 33% of the patients who have some opportunistic presentation [13]. This fact creates a challenging condition due to the difficult diagnosis because physicians are trained to diagnose parsimoniously, and multiple infections of the same anatomical site can simulate a single infection due to the similarity of the symptoms among them [14, 15]. Regarding the possible synergisms in the coinfection by different agents, bidirectional interaction of HIV and tuberculosis coinfection has already been described, such that one contributes to the aggravation of the other. HIV increases the risk of reinfection and reactivation of *M. tuberculosis* by reducing the Th1 response profile of the immune system. At the same time, tuberculosis in the patient with HIV promotes a reduction of CD4+ T cells and accelerates HIV replication through the production of tumor necrosis factor-alpha (TNF-alpha) and monocyte chemotactic protein 1 (MCP1) [14, 16].

We will discuss the correlation and immunological effects of HIV, *M. tuberculosis*, and CMV coinfection by reporting a case of tuberculosis in the ileocecal valve and descending colon and cytomegalovirus in the esophagus and descending colon, as well as with chronic hepatitis C virus (HCV) infection. The understanding of the behavior of the immune system in the face of simultaneous infections is a challenging scenario with scarce publications, especially about humoral and cellular immune response aspects.

## 2. Case Report

**Figure 1:**
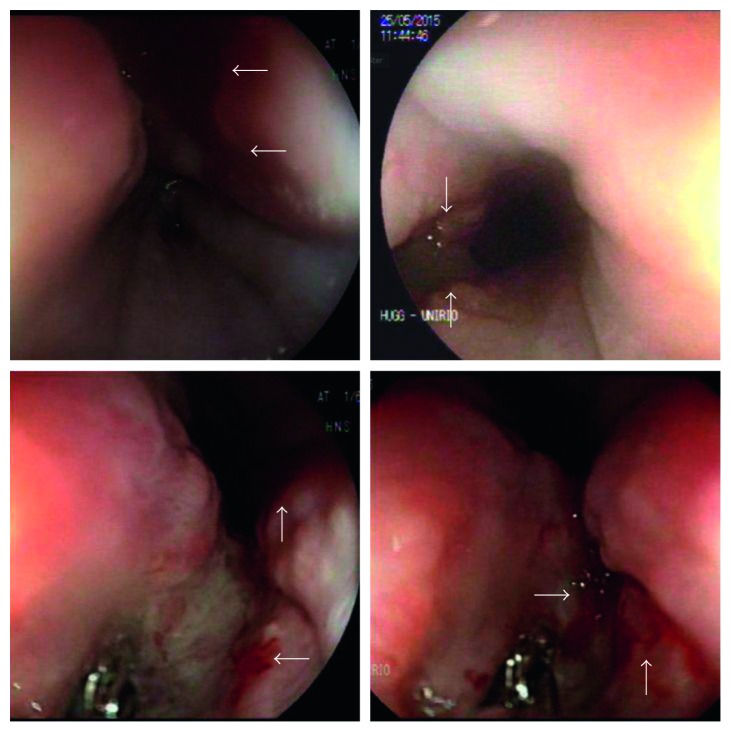
Upper digestive endoscopy showing an irregular ulcer with raised edges, fibrinolytic background, measuring approximately 3 cm in the middle third of the esophagus and 30 cm from the incisors and mild antrum gastritis.

**Figure 2:**
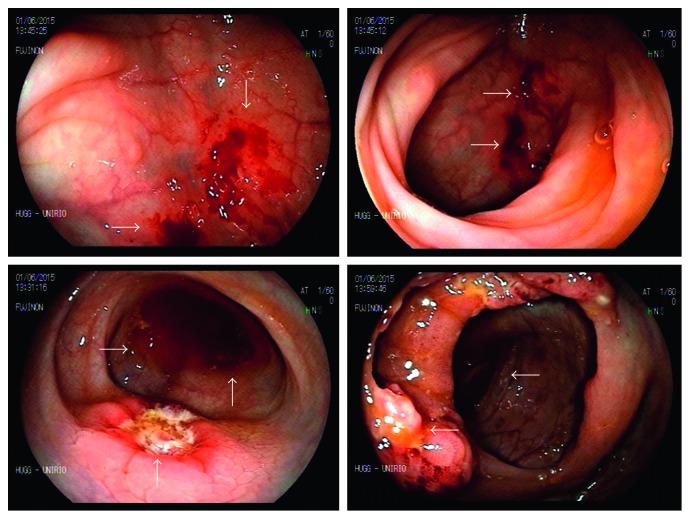
Colonoscopy showing ulcers with raised and irregular borders and necrotic background measuring between 1.5 and 2 cm and flat erosions.

**Figure 3:**
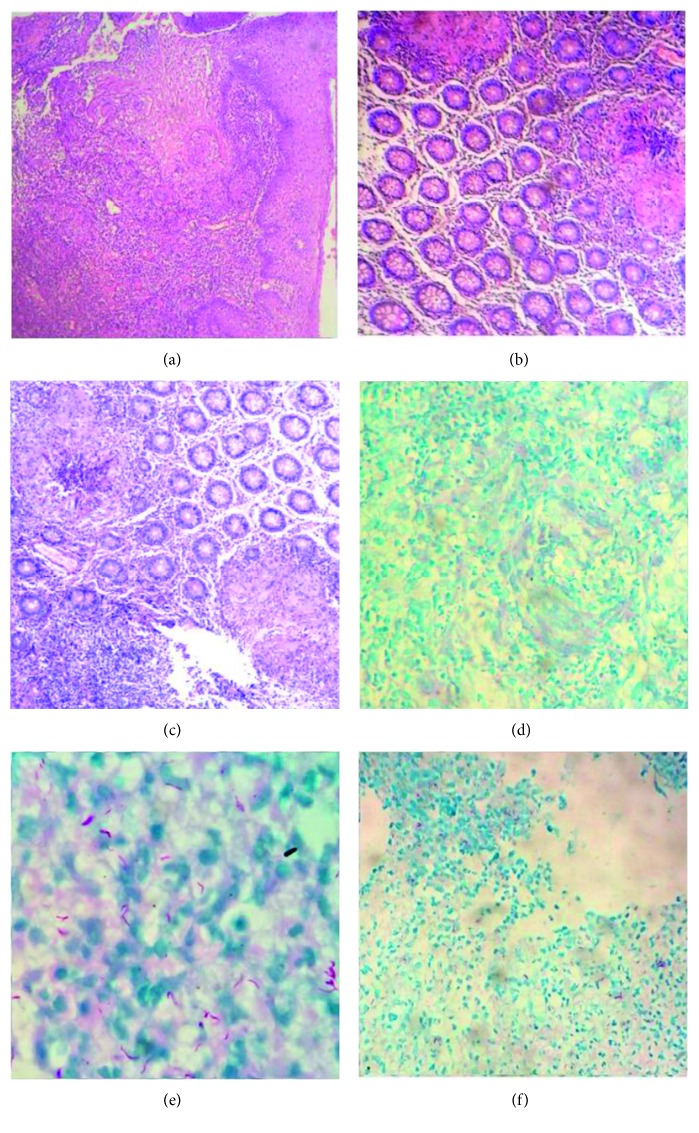
Esophagus (a, d), ileocecal valve (b, e), and colon (c, f). Histopathological examination showing chronic granulomatous inflammation with necrotic area and giant cells, at 200x magnification (a, b, and c), in addition to alcohol-acid-resistant bacilli by Ziehl–Neelsen staining, at 400x magnification (e). (d, f) The immunohistochemical study with positive labeling for CMV in cells with clear halos, at 400x magnification.

A 41-year-old man, a native from Rio de Janeiro and an HIV and HCV carrier, without criteria for the treatment for HCV (detectable viral load, without cirrhosis and with normal transaminase levels), who had abandoned ART, had attended the Gaffreé and Guinle University Hospital's immunology clinic complaining about continuous epigastric burning pain without irradiation and with diffuse abdominal pain that was mild and continuous, which had started approximately two months prior to admission. He also complained about intense hematochezia that had started three weeks before, with intense flow and with “pure blood” appearance without clots. He presented with daily hyperthermia since the abdominal symptoms had started with intermittent high fever and an over 10% body weight loss in the same period. The physical examination revealed oral candidiasis, bleached mucous membranes, and cachexia. At the admission time, the HIV viral load was recorded at 905,569 copies per ml, and the TCD4 lymphocyte count was 144 cells/dL. Prophylactic sulfamethoxazole-trimethoprim 400/80 mg 2 IV ampoules once daily and fluconazole 200 mg IV once daily for treatment of the oral candidiasis were prescribed. The patient's condition evolved without major occurrences or complaints, presented with high fever, above 38°C almost every day. Blood counts revealed thrombocytopenia, neutrophilia, lymphopenia, anemia, microcytosis, and anisocytosis (Table 1). The medical team requested upper digestive endoscopy (Figure 1) and colonoscopy (Figure 2), which verified the presence of ulcer with irregular and raised edges, fibrinonecrotic base, measuring approximately 3 cm in the middle third of the esophagus and 30 cm from the incisors and the mild antrum gastritis, and swollen, irregular, and fibrinous ulcers in the ileocecal valve, descending colon, and all other segments. The lesions were similar to those found in the esophagus, which could suggest the same etiology. It was suggested by the internal medicine team that the diagnosis could be a coinfection (tuberculosis, cytomegalovirus, and herpes simplex virus disease). The diagnosis of tuberculosis and cytomegalovirus coinfection of the gastrointestinal tract was confirmed by the histopathological report (Ziehl–Neelsen staining of acid-fast bacilli, CMV intracytoplasmic inclusions in Giemsa staining, and immunohistochemical study with positive labeling for CMV in cells with clear halos), and some time later, culture with the growth of *M. tuberculosis* (Figure 3). Treatment was started with an RIPE scheme (rifampicin + isoniazid + pyrazinamide + ethambutol) 4 tablets daily and ganciclovir 350 mg IV for 21 days with a weight gain of 4 kg and clinical and laboratory improvement. He was discharged from the hospital with ART lamivudine, tenofovir, and efavirenz (TDF + 3TC + EFV) one tablet per day and was referred to a clinical follow-up for tuberculosis and HIV/HCV coinfection monitoring. At the end of the treatment for tuberculosis and 6 months after ART was restarted, the patient's viral load was <40 copies/dL and the CD4+ T-cell count was 356 cells/dL, asymptomatic.

## 3. Discussion

In the present case, an AIDS patient with ART abandon, coinfected with HCV, tuberculosis, and simultaneous cytomegalovirus in the gastrointestinal tract, was reported. In the laboratory tests, microcytic anemia with anisocytosis suggested iron deficiency secondary to nutritional causes and chronic gastrointestinal bleeding. AIDS-typical laboratory findings were lymphopenia with neutrophilia and thrombocytopenia, which may also be related to chronic HCV infection [17].

CMV retinitis is the most frequent presentation of cytomegalovirus, accounting for up to two-thirds of the manifestations in target organs [18]. However, it is not uncommon to find extraocular manifestations in immunosuppressed patients, such as the pulmonary, neurological, and intestinal forms; the last one represents approximately 5 to 10% of the total [11, 18]. In gastrointestinal cytomegalovirus, colitis is the most common presentation, followed by esophagitis [19], occurring exactly at these sites in the case described. In tuberculosis cases, the most frequent form is pulmonary, and the extrapulmonary forms, less common and related to immunosuppression, represent only 14.4% of all diagnoses [1, 7]. From all the extrapulmonary forms, the intestinal is the sixth in frequency, and approximately 25–30% of the cases have the concomitant pulmonary form, which was not observed in our patient. The esophageal involvement here presented is rare and of lesser severity, representing only 0.15% of all deaths due to tuberculosis [9].

The tuberculosis and cytomegalovirus coinfection, although rarely described, has been already reported in other tissues [4, 20, 21]. In the gastrointestinal tract, it was described only once, in a patient with a recent diagnosis of HIV, affecting the distal ileum and ascending colon, presenting shock due to massive intestinal hemorrhage. As such complications are rare in intestinal tuberculosis, the hypothesis was raised that this complication could have been caused by a possible synergism between tuberculosis and cytomegalovirus, which was confirmed by histopathology [3]. However, the esophagus involvement by these two diseases together, as in the case here presented, has not been described yet.

The negative effect that HIV infection plays on the TH1 immune response profile, which is the main effective response against mycobacteria [16] and essential for maintaining CMV latency [4], is a determining immunological factor for this type of association [16]. Additionally, the cytopathic lesion that HIV itself produces in the intestinal epithelium is already a facilitator to new infections in this site [22]. This process occurs by the virus direct toxic action, by the lymphocytes inflammatory infiltrate in the lamina propria, and by the gastrointestinal immune system activation, with increased TNF-alpha production, a cytokine related to enterocytes apoptosis [23]. On the other hand, what we have observed is that the opportunistic infections themselves also contribute to the HIV progression. As HIV is predisposed to infect CD4+ T cells, any local infection that causes the recruitment of such cells will lead to more infection targets for the virus and its consequent replication [23]. Tuberculosis infection also promotes the abrupt decline in CD4+ T cells in HIV carriers, more specifically the Th1 cells. Additionally, increased viral replication has been demonstrated in those macrophages located in mycobacterial-infected sites, due to the increase of NF-kappa beta and TNF-alpha [15, 16]. Moreover, the relative risk of death and development of other opportunistic infections is greater in patients with HIV-TB coinfection [24].

Tuberculosis has also been demonstrated to influence CMV reactivation by inducing Mip-1beta production by CMV-specific response CD4 cells. Thus, tuberculosis infection also becomes a risk factor for CMV reactivation by accelerating the immunodepression progression by HIV. In addition, tuberculosis also stimulates the production of inflammatory cytokines that aid CMV reactivation [4].

As tuberculosis is an infection that manifests itself independently of CD4 T-cell levels [14], it is more likely to be the first opportunistic infection to occur and thus predispose the organism to others that occur with a lower CD4 count, such as CMV. However, as in the case described, the presentation of tuberculosis was intestinal, which depends on a more pronounced immunosuppression degree, and further without concomitant pulmonary infection, we cannot rule out the hypothesis that CMV would have appeared initially and, by the local replication of HIV itself, it would have caused tuberculosis reactivation at the same site.

Additionally, CMV actively infects monocytes, which are essential cells in the production of inflammatory cytokines that modulate the immune response to tuberculosis and HIV infection [11, 15]. It is therefore likely that CMV infection takes part in causing other infections or the reactivation of opportunistic agents due to inflammatory cytokine production, similar to what has already been described in tuberculosis and HIV. CMV also promotes a change in natural killer (NK) cell receptor patterns in the body with the predominance of the inhibitory-type NK cell receptors (NKG2C) compared to the activation type (NKG2A) [25], impairing the body's responses to other agents. The mechanisms described may partially explain the multidirectional interaction between these infections, so that only one of them itself is enough to cause the emergence of the others.

In the other case described in the literature [3], no clinical improvements were identified until CMV infection was identified and treated, which necessitates the skilled diagnosis of coinfections.  Table 2 compares clinical and epidemiological aspects between tuberculosis and CMV infection in the gastrointestinal tract, demonstrating their main differences [9, 16, 26, 27]. The lesions' appearances, found in our case's colonoscopy, were suggestive of ulcerative-type intestinal tuberculosis, but the significant ulceration extension that was found led to the suspicion of a coinfection responsible for magnifying the damage to the epithelium.

It should be noted that CMV has been reported in AIDS patients in coinfection with other diseases, such as pneumocystosis and *Mycobacterium avium* infection in the pulmonary tract [20, 21], and it has also been proposed as a facilitating agent for Kaposi's sarcoma [28]. Likewise, tuberculosis has been reported in coinfection with other opportunistic diseases, mainly in the pulmonary tract, such as pneumocystosis, histoplasmosis, *M. avium*, and paracoccidioidomycosis [2, 20, 21, 29, 30]. Possibly, the frequency of these coinfections mentioned above is greater than we imagine, especially in cases in which presentation occurs more severely than usual, with complications such as perforation, intestinal obstruction, or major hematochezia. These more severe presentations may indicate more than one agent in a synergistic interaction [3].

Thus, tuberculosis infection in the esophagus, a nonprevalent site for this infection, could have resulted from synergism with CMV causing an infection in a less probable site with increased ulceration. The hypothesis of coinfections is valid in less frequent and more aggressive presentations of opportunistic diseases.

## Figures and Tables

**Table 1:** Blood count on admission.

White blood cells (differential)	6.4 (10³/*μ*L) (0/0/0/0/11/65/15/9) (%)
Red blood cells	4.3 (10³/*μ*L)
Hemoglobin	10.8 (10³/*μ*L)
Hematocrit	33.3 (%)
MCV	77.4 (fL)
MCH	25.1 (pg)
MCHC	32.4 (g/dL)
Platelets	71 (10³/*μ*L)
RDW-SD	43.3 (fL)
RDW-CV	16.4 (%)
MPV	— (fL)
Neutrophils	6.18 (10³/*μ*L), 73.5%
Lymphocytes	1.5 (10³/*μ*L), 17.9%

**Table 2:** Main differences between intestinal cytomegalovirus and intestinal tuberculosis.

	Intestinal CMV	Intestinal tuberculosis
Lesion's features	Large and solitary ulcer or multiple ulcers, erosions, and mucosal hemorrhage	Solitary ulcer with an excavating base and rolled-up nodular edges
Main distribution in the gastrointestinal tract	Colon	Ileocecal valve
Gender	No predilection for gender	Male
Infection prevalence in HIV patients	3–5%	37%
Relation to immunosuppression degree	TCD4 <50 cells/dL	Any TCD4 cell counts
Main geographic distribution	South America, Asia, and Africa	South America, Asia, and Africa
